# Bone Impairment in Phenylketonuria Is Characterized by Circulating Osteoclast Precursors and Activated T Cell Increase

**DOI:** 10.1371/journal.pone.0014167

**Published:** 2010-11-30

**Authors:** Ilaria Roato, Francesco Porta, Alessandro Mussa, Lucia D'Amico, Ludovica Fiore, Davide Garelli, Marco Spada, Riccardo Ferracini

**Affiliations:** 1 Center for Experimental Research and Medical Studies, A.O.U. San Giovanni Battista, Torino, Italy; 2 Department of Pediatrics, University of Torino, Torino, Italy; 3 Department of Orthopaedics, A.O.U. San Giovanni Battista, Torino, Italy; Hungarian Academy of Sciences, Hungary

## Abstract

**Background:**

Phenylketonuria (PKU) is a rare inborn error of metabolism often complicated by a progressive bone impairment of uncertain etiology, as documented by both ionizing and non- ionizing techniques.

**Methodology:**

Peripheral blood mononuclear cell (PBMC) cultures were performed to study osteoclastogenesis, in the presence or absence of recombinant human monocyte-colony stimulating factor (M-CSF) and receptor activator of NFκB ligand (RANKL). Flow cytometry was utilized to analyze osteoclast precursors (OCPs) and T cell phenotype. Tumour necrosis factor α (TNF-α), RANKL and osteoprotegerin (OPG) were quantified in cell culture supernatants by ELISA. The effects of RANKFc and anti-TNF-α antibodies were also investigated to determine their ability to inhibit osteoclastogenesis. In addition, bone conditions and phenylalanine levels in PKU patients were clinically evaluated.

**Principal Findings:**

Several *in vitro* studies in PKU patients' cells identified a potential mechanism of bone formation inhibition commonly associated with this disorder. First, PKU patients disclosed an increased osteoclastogenesis compared to healthy controls, both in unstimulated and M-CSF/RANKL stimulated PBMC cultures. OCPs and the measured RANKL/OPG ratio were higher in PKU patients compared to healthy controls. The addition of specific antagonist RANKFc caused osteoclastogenesis inhibition, whereas anti-TNF-α failed to have this effect. Among PBMCs isolated from PKU patients, activated T cells, expressing CD69, CD25 and RANKL were identified. Confirmatory *in vivo* studies support this proposed model. These *in vivo* studies included the analysis of osteoclastogenesis in PKU patients, which demonstrated an inverse relation to bone condition assessed by phalangeal Quantitative Ultrasound (QUS). This was also directly related to non-compliance to therapeutic diet reflected by hyperphenylalaninemia.

**Conclusions:**

Our results indicate that PKU spontaneous osteoclastogenesis depends on the circulating OCP increase and the activation of T cells. Osteoclastogenesis correlates with clinical parameters, suggesting its value as a diagnostic tool for an early assessment of an increased bone resorption in PKU patients.

## Introduction

Phenylketonuria (PKU; OMIM 261600) is an inborn error of amino acid metabolism resulting from deficiency of phenylalanine hydroxylase, the key enzyme for phenylalanine metabolism [1]. An early protein-restricted diet integrated with phenylalanine free medical foods successfully prevents the irreversible developmental delay characteristic of the natural course of the disease, by maintaining plasma phenylalanine concentrations in non-neurotoxic range [2]. Despite the recommendation of life-long adherence to treatment, poor compliance to dietary prescriptions is common during adolescence [3], as the risk of mental retardation due to hyperphenylalaninemia was historically thought to be insignificant at this age. However, this laxity of dietary restriction, has been related to systemic complications of PKU in adulthood, as extensively reported recently [4]. Among these complications, bone impairment of uncertain etiology has been widely documented, using both radiological and ultrasound methods [5]–[9] and it is typically associated with increasing age.

Bone is a highly dynamic tissue undergoing continuous remodelling, with a fine equilibrium between bone formation by osteoblasts and resorption by osteoclasts (OCs) [10]. The relative preponderance of OC activity has recently been implicated in the pathogenesis of bone impairment in some conditions [11], leading to a growing interest towards OC biology and osteoclastogenesis [12]–[15]. Circulating OC precursors (OCPs) have been reported in several bone diseases, characterized by bone loss [16]–[18]. The main cytokines involved in the regulation of OC differentiation and function have been defined: macrophage colony stimulating factor (M-CSF) induces the proliferation and differentiation of OCPs; receptor activator of nuclear factor kB ligand (RANKL) promotes OC activity and decreases their apoptosis [19]; osteoprotegerin (OPG) is the RANKL neutralizing soluble decoy receptor [20]. An important link between immune system and bone has been established, with OCPs requirement of activated T cells to differentiate into OCs *in vitro*, in unstimulated conditions [13], [21], [22]. Our group previously demonstrated an increased spontaneous osteoclastogenesis in a small cohort of PKU patients [23], consistent with the increased bone resorption markers in affected patients [24]. In this study, we further investigated osteoclastogenesis in PKU, considering its potential causes and links with immune system; taking into account the individual bone condition assessed by phalangeal Quantitative Ultrasound (QUS).

## Results

### Osteoclastogenesis in PBMC cultures from PKU patients

**Figure 1:**
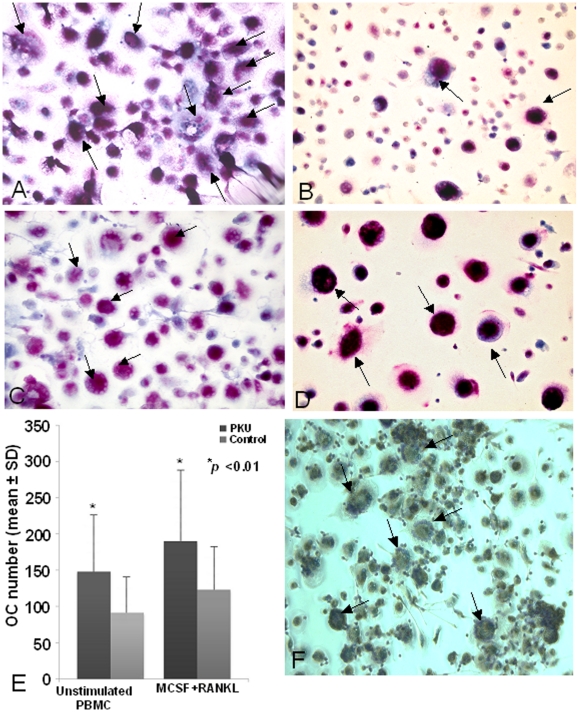
Osteoclastogenesis in PKU patients and healthy controls. Numerous, multinucleated (<3 nuclei/cell), TRAP+ OCs (black arrows) were obtained from unstimulated PBMCs of PKU patients (A), while few OCs were observed in healthy control cultures (B). After addition of M-CSF and RANKL, a significant increase in osteoclastogenesis was observed both in PKU patient and in healthy control PBMC cultures (C, D, respectively). The OC number in PBMC cultures was quantified, resulting higher in PKU patients than in healthy controls (E). OCs were normally distributed, hence PKU patients and healthy controls were compared by means of unpaired T-test. OCs from PKU patients' PBMC cultures expressed vitronectin receptor (F).

Numerous large tartrate-resistant acid phosphatase (TRAP) positive and multinucleated OCs were identified in the unstimulated PBMC cultures from PKU patients (OC average number/well 149±80; Fig. 1A), whereas few OCs appeared in the unstimulated PBMC cultures from healthy controls (OC average number/well 91±51; Fig. 1B). After M-CSF and RANKL addition to the cultures, osteoclastogenesis increased in the PKU patients (OC average number/well 189±97; Fig. 1C) and in healthy controls (OC average number/well 124±67, *p*<0.05; Fig. 1D). The osteoclastogenesis in PKU patients was significantly higher than in healthy controls, in unstimulated and stimulated cultures, *p*<0.01 (Fig 1E). OCs of PKU patients were then characterized for the expression of vitronectin receptor, a typical OC marker (Fig. 1F). PKU osteoclastogenesis depends on T cells, as in T cell-depleted cultures OCs did not differentiate without exogenous factors (data not shown). The bone resorbing activity was higher in unstimulated PKU patients' cultures than in healthy controls (Fig. S1).

### Circulating OCPs are increased in PKU patients

**Figure 2:**
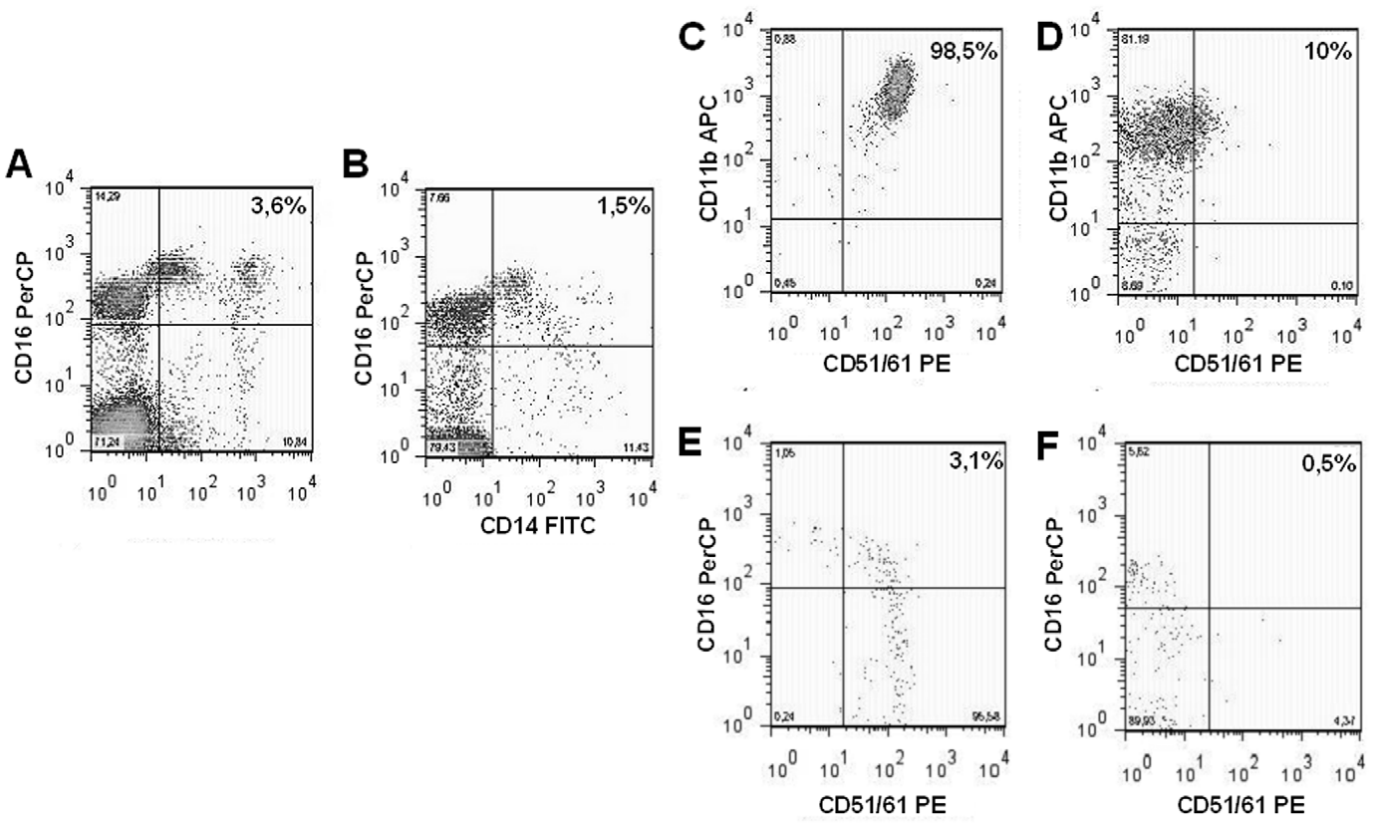
Circulating OCPs are increase in peripheral blood of PKU patients. Representative dot plots of CD14+CD16+ OCPs (A, B). The analysis of the expression of OCPs markers on CD14+ cells show CD11b+CD51/61+ (C, D) and CD16+CD51/61+ (E, F) increase in PKU compared to controls.

Based on CD16 expression and monocyte classification criteria set previously published [25], human CD14+ monocytes can be divided in two subsets CD16- and CD16+. CD14+ CD16+ can differentiate into OCs [26], and are classified as OCPs. In monocyte population, the number of CD14+ CD16+ OCPs was higher in PKU patients (7.2±0.5%, Fig. 2A) than in healthy controls (2.7±1.1%, Fig. 2B). Analysis of the expression of specific OCP markers on CD14+ cells showed higher co-expression of CD11b and CD51/61 in PKU patients (74.2±16.9%, Fig. 2C) compared to healthy controls (7.5±3.2%, Fig. 2D). CD51/61 expression was higher on CD16+ cells from PKU patients (4.8±1.4%, Fig. 2E) than healthy controls (0.4±0.6%, Fig. 2F).

### Spontaneous osteoclastogenesis is mediated by RANKL

**Figure 3:**
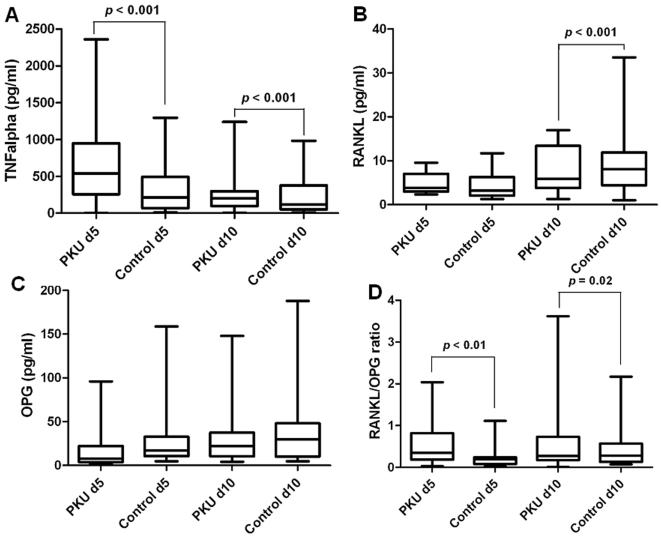
Osteoclastogenic cytokines in culture media. Box and whisker plots showed cytokines dosed in the PBMCs supernatants. Each Box represents the 25^th^ to 75^th^ percentiles. Lines outside the boxes represent the minimum and maximum values. Lines inside the boxes represent the medians calculated for all the data set. The *p* value indicated was calculated with the Mann-Whitney U test after correction for age. In PKU patients TNF-α was higher than in healthy controls (A). RANKL resulted significantly higher in PKU than in controls at day 10 (B), whereas OPG did not differ between patients and controls (C). The RANKL to OPG ratio was in favour of RANKL in PKU patients compared to healthy controls (D).

In order to identify molecules promoting osteoclastogenesis in cultures, we dosed TNF- α, RANKL and OPG in supernatants, at day 5 and 10 of culture. At day 5, TNF-α levels were higher than at day 10 and its concentration increased dramatically in patients' cultures compared to healthy controls (Fig. 3A, *p*<0.001). At day 10, RANKL release resulted significantly increased in PKU patients compared to healthy controls (Fig. 3B, *p*<0.001). OPG levels in PKU patients and healthy controls were not significantly different (Fig 3C). However, the RANKL/OPG ratio was significantly higher in PKU patients than in healthy controls (Fig. 3D, *p*<0.01), explaining the presence of spontaneous osteoclastogenesis in unstimulated cultures of PKU patients.

**Figure 4:**
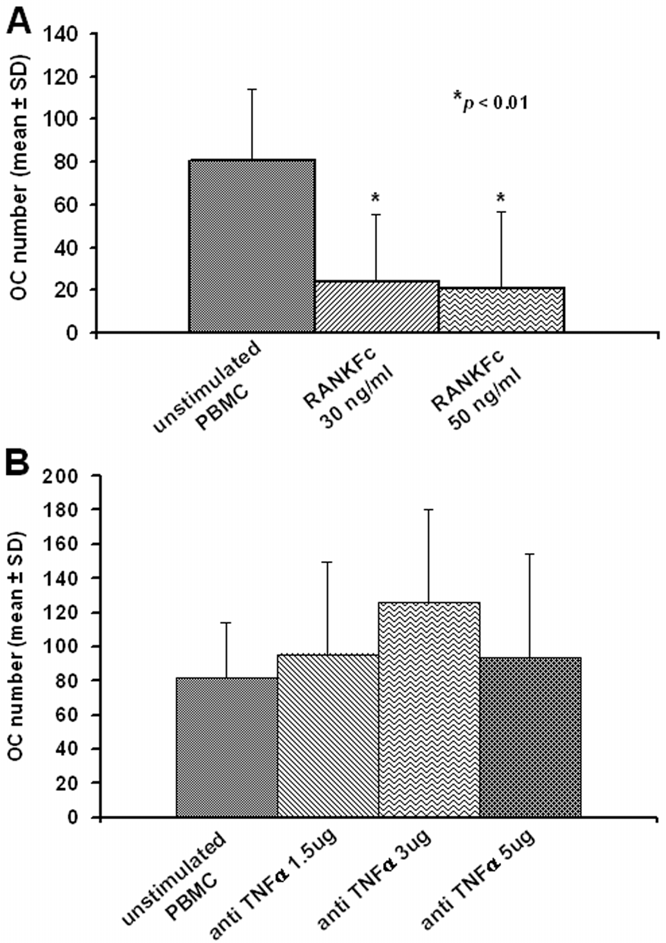
PKU osteoclastogenesis is RANKL-dependent. The RANKFc addition to unstimulated PBMC culture from PKU patients caused a dose-dependent inhibition of OC formation (A), whereas anti-TNF-α failed to inhibit osteoclastogenesis (B). The *p* value indicated was calculated by unpaired T-test.

To define the main molecules that promote osteoclastogenesis, we added RANKFc and anti-TNF-α at different concentrations to patient PBMC cultures. A marked and significant osteoclastogenesis inhibition was detected by RANKFc compared to unstimulated PBMC cultures, where OCs spontaneously differentiate (Fig. 4A, *p*<0.01). Anti-TNF-α failed to significantly reduce osteoclastogenesis compared to the unstimulated condition (Fig. 4B).

### PKU patients show an increase number of circulating activated T cells

**Figure 5:**
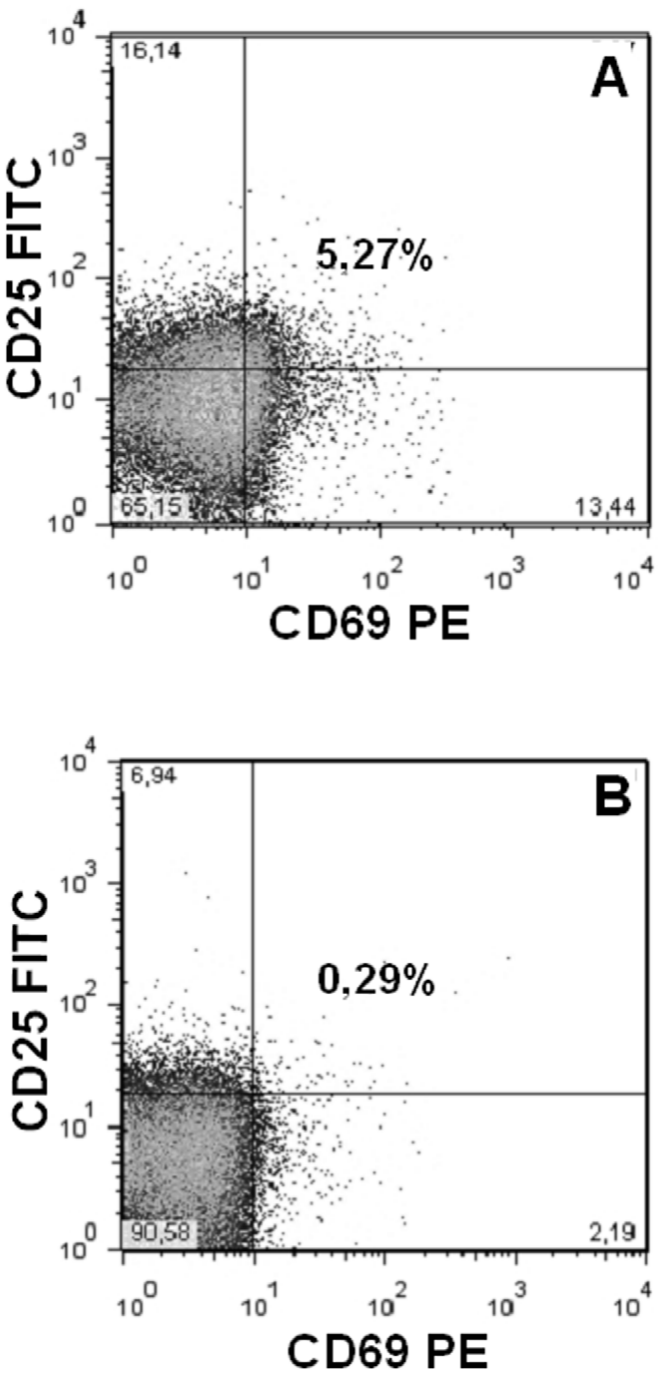
PKU patients have activated T cells in peripheral blood. Representative dot plots of CD4+ T cells expressing both CD69 and CD25 in PKU patient (A) and healthy controls (B).

T cell phenotype from PKU patients were analyzed to investigate their possible involvement in promoting osteoclastogenesis, as described in many diseases characterized by bone loss. Other than expressing RANKL (data not shown), we found that T cells also expressed CD69 and CD25, two typical activation markers. CD4+ cells were significantly reduced in PKU patients compared to healthy controls (67.3±7.1% and 87.6±10.2%, respectively, *p*<0.01). 3.7%±1.1 of CD4+ cells, expressed both CD69 and CD25 in PKU patients (Fig. 5A), whereas healthy control CD4+ cells did not co-express the two markers (Fig. 5B). CD8+ cells were comparable in patients and controls (27.5±11.9% and 31±5.3%, respectively). The number of activated T cells was directly correlated with osteoclastogenesis (r = 0.483, *p*<0.001), and inversely correlated to QUS parameters (AD-SoS SDS: r = −0.210, *p*<0.05; BTT SDS: r = −0.292, *p*<0.05).

### Spontaneous osteoclastogenesis correlates with clinical PKU parameters

**Figure 6:**
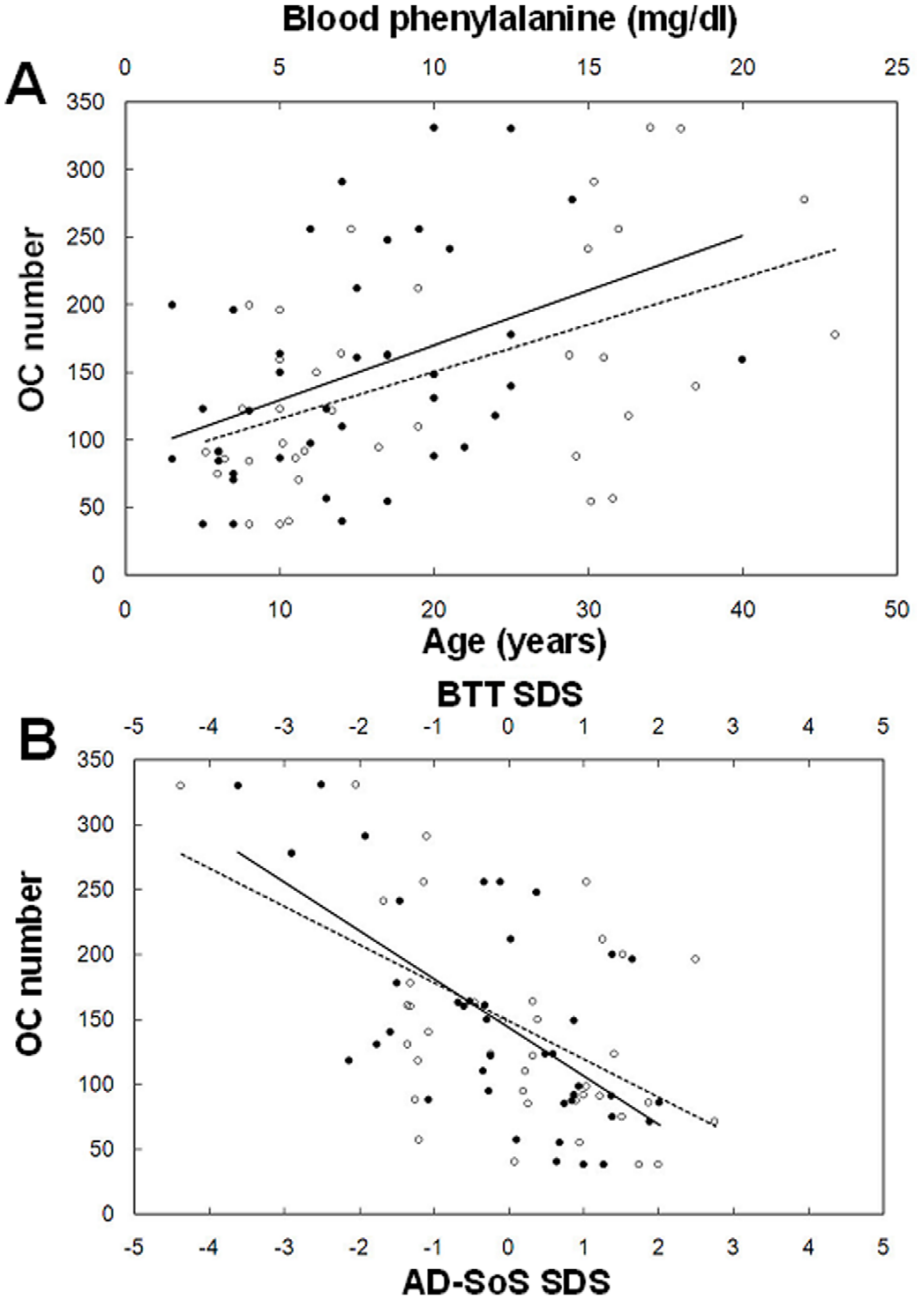
PKU osteoclastogenesis correlates with clinical parameters. Spontaneous osteoclastogenesis in PKU patients shows a significant correlation with age (•, continuous line) and blood phenylalanine concentration, dosed in the last year before the study (○, dotted line). PKU patients showed a significant negative correlation between spontaneous osteoclastogenesis and bone condition assessed by QUS parameters, Amplitude-Dependent Speed of Sound (AD-SoS) (○, dotted line) and Bone Transmission Time (BTT) (•, continuous line). Correlations were evaluated by Pearson's coefficients.

In PKU patients, spontaneous osteoclastogenesis was directly correlated with both age (r = 0.386, *p* = 0.03) and blood phenylalanine concentration (r = 0.458, *p* = 0.01), and these correlations were not observed in healthy controls (Fig. 6A). Moreover, PKU patients displayed a significant negative correlation between QUS parameters and spontaneous osteoclastogenesis (Amplitude-Dependent Speed of Sound, AD-SoS SDS: r = −0.553, *p*<0.001; Bone Transmission Time, BTT SDS: r = −0.631, *p*<0.001) (Fig. 6B).

QUS assessment in PKU patients revealed an overall normal bone condition comparable to healthy population (AD-SoS SDS = 0.02±1.51, *p* = 0.946; BTT SDS = −0.13±1.35; *p* = 0.550). Fourteen patients (35%, mean age 22.4±6.6 years) had abnormal bone scans, with a reduction of both parameters with respect to normal population (AD-SoS SDS  = −1.61±0.97, *p*<0.001; BTT SDS  = −1.60±0.98; *p*<0.001). The 22 patients aged <15 years (mean age 8.7±3.6 years) showed increased AD-SoS and BTT with respect to normal population (AD-SoS SDS  = 0.88±1.03, *p*<0.001; BTT SDS  = 0.62±0.93; *p*<0.001). Similarly, bone impairment was also observed in the 18 patients aged >15 years, (AD-SoS SDS  = −1.04±1.34 and BTT SDS  = −1.04±1.24; both parameters, *p*<0.001). In patients with blood phenylalanine level steadily lower than 10 mg/dl (n = 24, mean age 10.8±7.8 years), AD-SoS and BTT SDS were significantly higher than controls (0.93±0.92 and 0.62±0.78, respectively, *p*<0.001), whereas reduced QUS parameters were observed in patients with phenylalanine concentration >10 mg/dl (n = 16, mean age 20.3±4.5 years, AD-SoS SDS  = −1.35±1.16, *p*<0.001; BTT SDS  = −1.26±1.26; *p*<0.001). In addition QUS parameters were negatively correlated with both patients' age (AD-SoS SDS: r = −0.736, *p*<0.001; BTT SDS: r = −0.726, *p*<0.001) and mean blood phenylalanine concentration (AD-SoS SDS: r = −0.788, *p*<0.001; BTT SDS: r = −0.807, *p*<0.001).

## Discussion

In the field of biochemical genetics, there has been a growing interest towards adult complications of PKU [4]. In particular, age related bone impairment has been widely reported in PKU patients, mainly by using radiological methods (5–8). To date, the “gold standard” for bone assessment in childhood is still debated [27]. Currently dual x ray absorptiometry (DXA) remains the most commonly employed method for bone assessment. However, this technique may present some limitations when applied in the pediatric age group. In addition to the repeated radiological exposure to adolescents, DXA provides a selective estimation of bone mineral density on the basis of a two dimensional measurement dependent on the bone surface area; a finding largely influenced by the physiological modifications of bone geometry occurring during growth [28]. Consequently, QUS methods have been increasingly utilized for bone assessment at different ages, including young patients [9]. These techniques are based on the principle that ultrasound is differently modified in its shape, intensity, and speed on the basis of structural properties of bone [29]. QUS also provides additional information compared to DXA, such as cortical thickness, porosity, elasticity, and anisotropy [30]–[32]. Prior reports have demonstrated the strict correlation of phalangeal to the gold standard bone mineral density measurement by DXA. In particular, significant correlation between both spine- and total body-bone mineral density assessed by DXA and phalangeal QUS has been reported (r = 0.45 p<0.05 and r = 0.56, p<0.01, respectively) [33]. Moreover, phalangeal QUS is particularly suitable for bone evaluation in pediatrics, as it is easy and rapid to use, non-invasive, inexpensive, and radiation-free [34]. Whatever the method employed for bone assessment, the relationship among the bone impairment, the effects of early protein restriction, and the commonly observed late dietary discontinuation with consequent chronic hyperphenilalaninemia is not completely clarified [23]. However, a pathogenic role of chronic exposure to high blood phenylalanine concentration on the bone compartment has been described in animal models [26]. Bone impairment in PKU seems to reflect the relative preponderance of OC activity, with disruption of the physiological homeostatic equilibrium between bone formation and resorption [35]. Considering the central role of OCs in the pathogenesis of diseases characterized by increased bone resorption, the investigation of osteoclastogenesis from PBMCs may be a promising approach to understand the mechanism of bone loss in several conditions, as recently outlined in different diseases, including PKU [11], [17], [23].

A prior study from our group [23], reported a higher incidence of spontaneous osteoclastogenesis (PBMCs differentiating into OCs without exogenous factors) in a large cohort of PKU patients compared to healthy controls. Additionally, we previously reported that supplementation of M-CSF and RANKL led to an increase of osteoclastogenesis in PKU patients and in healthy controls, indicative of a physiological response to stimulation. This work aimed to expand on that study by investigating the causes of the osteoclastogenesis. This was performed by studying the presence of an abnormal number of circulating OCPs, since one cause of spontaneous osteoclastogenesis in different pathological conditions characterized by bone loss is represented by their increase [36]. First the number of OCPs expressing CD14/CD51-61/CD11b and a particular OCP subset, expressing CD14 and CD16, were elevated in PKU. This subset of OCPs expressing CD14 and CD16 was previously described in inflammatory diseases characterized by loss of bone mass, such as psoriatic arthritis [25]. In order to identify the molecules responsible for osteoclastogenesis in unstimulated PBMC cultures from PKU, we measured the TNF-α, RANKL and OPG concentrations in supernatants at different time points. TNF-α concentrations were higher in cultures from PKU patients than in healthy controls. Intersestingly, TNF-α levels were particularly high at the beginning of the culture, suggesting a TNF-α involvement in promoting OCPs, which self-regulate TNF-α release. In fact, CD14+CD16+ OCPs are a major source of TNF-α [37] and CD16 regulates both the TNF-α activation and inhibition. We hypothesize that the TNF-α variation at different time points may be due to CD16 regulation, where CD16 increases and then inhibits the TNF-α release. The RANKL to OPG ratio was higher in PKU patients than in healthy controls, suggesting the role of RANKL as major promoter of the osteoclastogenesis in PKU patients. To confirm this result, we showed OC inhibition after RANKFc addition to unstimulated PKU cultures. On the contrary, we did not observe any osteoclastogenesis modulation after addition of the anti-TNF-α antibody, confirming our hypothesis that TNF-α acts on OCPs. The osteoclastogenesis dependence on T cells has been widely demonstrated in many diseases characterized by increase bone resorption activity [22], [38]–[40]. In this study we identified such a mechanism in PKU osteoclastogenesis, by the lack of OC differentiation in T cell-depleted culture, without addition of exogenous factors. This result is in accordance to prior findings and can be explained by RANKL expression on T cells. The number of CD4+ T cells in PKU patients was reduced compared to healthy controls. However PKU patient CD4+ T cells expressed CD69 and CD25, typical activation markers. Moreover, the number of activated CD4+ T cells directly correlated with osteoclastogenesis, confirming the role of T cells in promoting OC differentiation. These data highlight a peculiar condition of immune system in PKU patients, which needs further investigations for its potential clinical relevance. Even though our group previously reported the presence of spontaneous osteoclastogenesis in PKU patients, this work describe a novel link between osteoclastogenesis and the immune system in the pathogenesis of bone impairment in PKU.

Osteoclastogenesis and the number of activated T cells in PKU patients were inversely related to bone condition. This finding suggests a possible relationship among spontaneous osteoclastogenesis, immune system and bone damage, as previously described in different diseases by our group and others [16], [18], [36]. In addition to the confirmation of the previously reported correlation between QUS parameters and metabolic control in PKU [9], a direct connection between plasma phenylalanine concentration and spontaneous osteoclastogenesis was detected. This is consistent with a possible role of hyperphenylalaninemia in enhancing OC differentiation and consequently promoting bone resorption.

In conclusion, PKU patients showed increased osteoclastogenesis compared to healthy controls, depending on an OCP increased number. RANKL regulates PKU osteoclastogenesis, whereas TNF-α seems to stimulate and be regulated by OCPs. The osteoclastogenesis and T cell activation state correlates with PKU patients' bone condition. Thus, the finding of a specific sub-population of activated T cells accounting for spontaneous osteoclastogenesis infers a dysfunctional immune system activation in PKU patients. We believe that the immune system in PKU patients needs to be deeply investigated for its potential clinical relevance.

## Materials and Methods

### Subjects

The study was approved by the Ethical Committee of Children Hospital Regina Margherita- S.Anna. Written informed consent was obtained from all subjects or from their parents when under age. Forty patients affected by PKU (18 males and 22 females, mean age 14.6±8.1 years) and 40 age- and sex-matched healthy controls were enrolled in this study. Height and pubertal development were assessed according to Tanner's standards and criteria. BMI was calculated using the weight/height^2^ (Kg/m^2^) formula, corrected for sex and age, and expressed as BMI SDS, according to Italian reference charts [41]. Exclusion criteria were the following: short stature (defined as height below -2 standard deviation for age), history of immobility or high physical activity, treatment with drugs interacting with bone metabolism (including calcium and vitamin D supplementations), sub-optimal nutrition (defined as dietary restriction with caloric intake less than 70% of recommended values for age or loss of more than 10% of body weight during the last year) and concomitant diseases with bone or blood involvement. Clinical features of patients and controls are detailed in table 1. PKU patients were treated with a protein-restricted diet supplemented with the same phenylalanine-free amino acid mixture in three portions during the day, warranting the daily recommended age-related intakes of macronutrients, minerals and vitamins [42]. For each patient, monthly plasma phenylalanine levels were collected in the year prior to the study. Healthy subjects enrolled as controls had a normo-caloric free diet, meeting the daily age-related recommendations [43].

**Table 1:** Clinical features of patients with phenylketonuria (PKU) compared to healthy controls.

CHARACTERISTICS	PKU	CONTROLS	*p*
**Number**	40	40	-
**Gender (Male/Female)**	18/22	18/22	-
**Age (years)**	14.6±8.1	12.2±6.3	0.15
**Height (cm)**	141.9±25.9	141.2±25.4	0.09
**BMI SDS**	−0.01±1.14	0.12±0.66	0.45
**Blood phenylalanine (mg/dl)**	9.8±5.8	0.9±0.3	<0.01

### Cell cultures

PBMCs were obtained from peripheral blood samples according to the Ficoll method, as previously described [18]. For all patients and controls PBMCs were plated in 24-well plates (2×10^6^ cells/well), using alpha-minimal essential medium (α-MEM, supplied by Invitrogen, Carlsbad, CA), supplemented with 10% fetal bovine serum, benzylpenicillin (100 IU/ml) and streptomycin (100 mg/ml) (supplied by Lonza, Basel, Switzerland) and maintained at 37°C in a humidified atmosphere of 5% CO_2_. To obtain fully differentiated human OCs, PBMCs were cultured in presence or absence of recombinant human M-CSF (25 ng/ml) and RANKL (30 ng/ml), for 15 days. In 7 independent experiments, PBMCs were cultured in 96-well plates (5×10^5^ cells/well) in the presence of increasing concentration of RANKFc (30–50 ng/ml) and anti-TNF-α (1,5–3–5 µg/ml) (supplied by PeproTech, London, UK). At the end of the culture period, cells were stained for tartrate-resistant acid phosphatase (TRAP, kit supplied by Sigma-Aldrich, St Louis, MO) and vitronectin receptor (Chemicon International, Temecula, CA). OCs were identified as TRAP positive, multinucleated cells, containing three or more nuclei. The T cell depletion was performed according to a previously published method [35]. To study OC resorbing activity, PBMCs from 5 patients and 5 healthy controls were plated on BioCoat osteologic bone cell culture system (5×10^5^ cells/well) provided by BD Biosciences (Bedford, MA) and cultured for 20 days. In order to visualize pits formed by OCs, the cells were removed by washing each well with NaOCl and resorption lacunae were identified by light microscopy. The quantification of resorbing area was perfomed by a semi-automated image analyzing system [44].

### Flow cytometry

To identify OCPs, aliquots of 1×10^6^ PBMC from 15 patients 15 controls were incubated with the following anti-human antibodies: APC-conjugated CD11b, PE-conjugated CD51/61 (BD Pharmingen, San Diego, CA), FITC-conjugated CD14 (Chemicon International, Temecula, CA) and PerCP-conjugated CD16 (Biolegend, San Diego, CA). To investigate the phenotype of T cells, PBMCs were stained with the following anti-human antibodies: APC-conjugated CD4, FITC-conjugated CD25 (Caltag, Burlingame, CA), PE-conjugated CD69 (Biolegend, San Diego, CA) and related isotype controls. The expression of RANKL by T cells was assessed by indirect staining using a monoclonal mouse anti-RANKL (Santa Cruz Biotechology, Santa Cruz, CA) as primary antibody and a goat anti-mouse FITC-IgG1 (SouthernBiotech, Birmingham, AL). Appropriate controls were used to determine optimal voltage settings and electronic subtraction for the spectral fluorescence overlap correction. Samples were analyzed in a FACsCalibur instrument (Becton Dickinson, Bedford, MA) and elaborated by Flowjo (Treestar, Ashland, OR).

### ELISA (Enzyme-Linked Immunosorbent Assay)

Supernatants from cultures not supplemented with growth factors were collected at day 5 and 10 (when medium was refreshed), and the concentrations of TNF-α (Quantikine; R&D Systems, Minneapolis, MN), RANKL (Biomedica; Biomedica Medizinprodukte GmbH and Co. KGA) and OPG (BenderMedSystems, Vienna, Austria) were measured by commercially available ELISA kit, according to manufacturer's instructions. The absorbance was determined by an ELISA reader at 450 nm and the results were expressed as mean ± SD.

### Assessment of bone condition

All patients underwent a phalangeal QUS measurement using the DBM Sonic-Bone Profiler (Igea, Carpi, Modena, Italy). The device consists of an electronic calliper with emitter and receiver probes recording ultrasound beam modifications through the phalanx. Acoustic coupling was achieved through standard ultrasound gel. Amplitude-Dependent Speed of Sound (AD-SoS) was measured in m/s, considering the first received signal with amplitude of 2 mV. AD-SoS is primarily related to the bone density [45] and low AD-SoS is associated with a reduced bone mineral density assessed by DXA. This parameter is influenced by the thickness of the surrounding soft tissues at proximal phalanges of the hand. Bone Transmission Time (BTT) was calculated in µs by subtracting the instant corresponding to the arrival time of the fastest signal from the time of transmission of a pulse at 1700 m/s velocity, being independent of soft-tissue thickness [46], [47]. BTT is closely related to cortical width and can discriminate different bone disease patterns independent of bone density [30].

All measurements were performed by the same trained operator at the distal metaphyses of the proximal phalanges of fingers II-V of dominant hand.

AD-SoS and BTT values were compared to sex- and age-matched healthy controls [47] and expressed as SDS. Short-term precision was assessed based on 100 measurements repeated on 20 healthy subjects aged 3–18 years measured 5 times each, disclosing intra-operator coefficients of variation of 0.72% and 1.07% for AD-SoS and BTT, respectively.

### Statistical analysis

The normal distributions of each parameter were determined by Kurtosis's test. Since none of the cytokines analyzed were normally distributed, TNF-α, RANKL and OPG were compared by means of the Mann-Whitney U test after weight for age. The other parameters showed a normal distribution and they were compared by Student's unpaired T test. Pearson's correlation coefficients were used to check univariate associations. The SPSS 17.0 software package was used to process the data with *p*<0.05 as the significance cut-off.

## Supporting Information

Figure S1 Bone resorption assay. Numerous resorption lacunae were observed in PKU patients compared to healthy controls (A, B, respectively). Bone resorbing activity resulted higher in PKU than in control (C). Magnification 20X.
(0.16 MB TIF)
