# Supplementary figures and images for: Bone Impairment in Phenylketonuria Is Characterized by Circulating Osteoclast Precursors and Activated T Cell Increase
Source: PLoS One. 2010 Nov 30;5(11):e14167. doi: 10.1371/journal.pone.0014167 (PMC2994752; doi:10.1371/journal.pone.0014167)

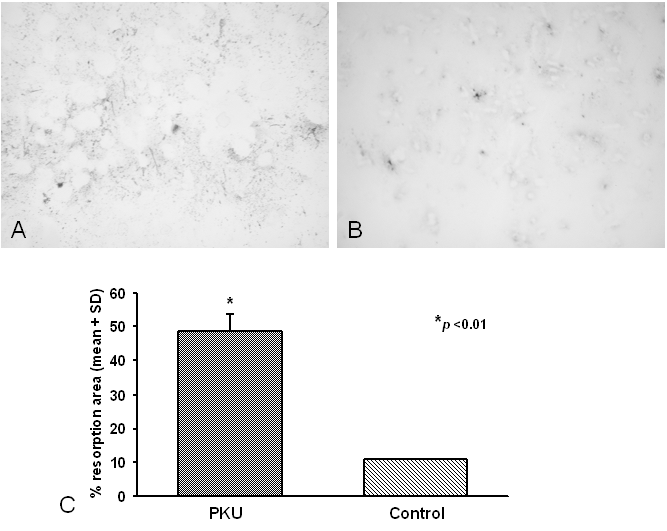

Supplement: Figure S1 — Bone resorption assay. Numerous resorption lacunae were observed in PKU patients compared to healthy controls (A, B, respectively). Bone resorbing activity resulted higher in PKU than in control (C). Magnification 20X. (0.16 MB TIF) [file pone.0014167.s001.tif]
